# The Vulnerability Typology: Developing a Biopsychosocial-Sexual Understanding of Men With Sexual Interests in Children

**DOI:** 10.5964/sotrap.13925

**Published:** 2025-07-01

**Authors:** Hannah Stewart, Mary Ann Campbell

**Affiliations:** 1 Department of Psychology, University of New Brunswick, Fredericton Campus, Fredericton, NB, Canada; 2 Department of Psychology, University of New Brunswick, Saint John Campus, Saint John, NB, Canada; Saint Mary's University, Halifax, NS, Canada

**Keywords:** pedophilia, hebephilia, prevention, minor attracted person, typology, sexual interest in children, men with sexual interest in children

## Abstract

The goal of the current study was to better understand biopsychosocial factors related to men with sexual interests in children (MSICs) who have no criminal history of sexual offending to clarify heterogeneity and potential targets for preventative intervention for persons who are at risk of sexual offending. Using online recruitment methods, self-report data on biopsychosocial-sexual factors was collected from community men reporting paraphilic and atypical sexual interests, including men with sexual interests in children (*n* = 609; *M*_age_ = 29.7 years) and a comparison group with other paraphilias (*n* = 224; *M*_age_ = 35.3 years). Data were used to develop a biopsychosocial-sexual typology of community men with pedohebephilic interests with no reported offence history. Latent cluster analysis identified three groups differentiated by relative endorsement of biopsychosocial vulnerability characteristics. Comparisons between profiles indicated a generally unimpaired profile (i.e., Low Vulnerability) relative to a group with intermediate vulnerabilities (i.e., Moderate Vulnerability) and a group with significant impairment across most assessed constructs (i.e., High Vulnerability). Results inform areas of need for secondary prevention among community MSICs with no historical justice system contact related to their sexual interests in children. Consistent with Risk-Need-Responsivity Model, this research suggests that intensity and dosage of preventative intervention for MSICs should match level of vulnerability.

## Highlights

- Community MSICs with no criminal justice history constitute distinct, heterogeneous groups.
- Vulnerability Typology can differentiate MSIC profiles based on biopsychosocial-sexual factors.
- Low Vulnerability MSICs are low need, but Moderate and High Vulnerability MSICs have elevated need.
- Per Risk-Need-Responsivity, level of vulnerability may guide intensity of prevention intervention.

Many terms have been used to refer to people who experience sexual interest in pre-pubescent (i.e., typically 13 years or younger, or “pedophilia;” American Psychological Association [APA], 2013) or pubescent (i.e., typically between 11-14 years, or “hebephilia;” Seto, 2017; Stephens et al., 2018) children who are under the age of consent (i.e., typically 16 years old or younger in North America legislation). Some terms have stigmatizing connotation and conflate constructs of sexual interest with sexual behaviour, such as “pedophile,” “child molester,” and “child sex offender.” More recently, terms like “minor attracted person” (MAP; B4U-ACT, 2014) emerged as alternative labels for more explicit separation between sexual interest and sexual behaviour, with arguably less association with prejudice or clinical pathology (Levenson & Grady, 2019). However, these terms have been criticized as being imprecise and inconsistent with efforts for using person-first language when describing subpopulations.

For clarity, the current research defined the terms “children” or “minors” as being ≤ 14 years old for consistency with ratings of sexual maturity in the physiological developmental features of pre-pubescent and pubescent children (e.g., Tanner Stages/Sexual Maturity Rating; Emmanuel & Bokor, 2022). The target population for the current study are “men with sexual interest in children” (MSIC) or who self-report some degree of “pedohebephilic” interests. The exception to this conceptualization applied to data on self-reported history of charges/convictions for child sexual abuse (CSA) offences, which used a cut off of ≤ 16 years old for consistency with legislative definitions in North America for non-consensual (i.e., illegal) sexual activity.

Having pedohebephilic interest is not illegal, but engaging in sexual activities with children who are below the legal age to provide consent is illegal (Fedoroff, 2018). This distinction is clear in that approximately 50% of CSA perpetrators experience sexual interests in children and have pedohebephilic sexual interest (i.e., experience sexual interest to pubescent and/or pre-pubescent children; e.g., Seto, 2018; Tenbergen et al., 2015). However, pedohebephilic interest may increase inclination to seek out situations involving children. Since pedophilia and hebephilia tend to be stable over time and act as meaningful CSA risk factors (e.g., Hanson & Morton-Bourgon, 2005; Seto, 2018; Stephens et al., 2024; Whitaker et al., 2008), interventions promoting effective management of pedohebephilic interests (e.g., behavioural or psychopharmacological; McPhail & Olver, 2020) may support risk management by reducing the likelihood of acting on these interests to victimize children.

Given the prevalence and effects of CSA, much research has investigated causes of sexual offending, identifying factors which impact sexual recidivism among criminal populations (Klein et al., 2018). Research with justice-involved men convicted of CSA provide frameworks and typologies for understanding dynamic interactions among internal and external factors in the etiology of and diverse pathways to CSA (Ward & Beech, 2008; Ward & Siegert, 2002). However, more research is needed to understand how models apply to people in the general population who might have elevated vulnerability for sexually offending against children, such as men who experience sexual interests in children but who have not acted of these interests in criminal ways. To clarify differences between sexual interests and sexual behaviours, the current research examined the profiles of community MSICs and examined how these subgroups compare to men with other paraphilias and a sub-sample of MSICs who report offending with CSA. Such insight can inform areas pertinent to the development of prevention, interventions, and best practice policies to reduce rates of child victimization.

## Pedohebephilic Interest Without Sexual Offending

Emerging research on people with pedohebephilic interests suggest disparities in neurological, psychological, social, and behavioural characteristic between people with pedohebephilia who have sexually offended against children versus those who have not (e.g., Beier et al., 2009; Gerwinn et al., 2018; Kärgel et al., 2015, 2017; Konrad et al., 2017; Massau et al., 2017; Schiffer et al., 2017; Stephens et al., 2024). For example, compared to non-justice involved MSICs, MSICs with CSA histories tend to display diminished functional activity in neural networks implicated in motivational and socio-emotional processes, socio-cognition (e.g., deciphering social cues, theory of mind, moral judgements, sexual disinhibition), and impulse inhibition (Kärgel et al., 2015, 2017; Massau et al., 2017; Schiffer et al., 2017). Gerwinn and colleagues (2018) also found associations with experience of CSA as a factor associated with perpetration of sexual offending against children more broadly, as well as high prevalence (45% to 58%) of mental illness relative to non-justice involved persons. Thus, MSICs who have perpetrated CSA represent a heterogeneous population compared to those with no criminal justice involvement.

Recent research examining detected and undetected MSICs by Stephens and colleagues (2024) suggested that clinical and risk factors such as additional paraphilias, hypersexuality, dark personality traits, and features of their pedohebephilic sexual interests and history of adverse childhood experiences differentiated those MSIC with CSA offences from those without detected CSA histories. Therefore, psychological risk factors may be particularly relevant for distinguishing MSICs who perpetrate CSA from those who do not. Furthermore, people with sexual interests in children tend to experience psychological distress and stigmatization, even if they have never perpetrated CSA (e.g., Beier et al., 2009; Gerwinn et al., 2018; Konrad et al., 2017; Lievesley et al., 2020). Unfortunately, this social and internalized stigma are deterring factors for help-seeking among MSIC populations, regardless of detection status (Elchuk et al., 2022; Houtepen et al., 2016; Jahnke, Imhoff, et al., 2015; Jahnke, Schmidt, et al., 2015; Lasher & Stinson, 2017; Levenson et al., 2017). A lack of access to professional resources may exacerbate existing psychological vulnerability and thereby increase an individual’s risk for perpetrating CSA and decrease their quality of life (B4U-ACT, 2011; Farmer et al., 2012).

## Typologies in Forensic Samples of Child Sex Offenders

Researchers have attempted to understand the diversity among men who perpetrate CSA by developing psychometric typologies to represent subtypes within the larger group of known offenders using risk factors and offence history variables (e.g., Robertiello & Terry, 2007). These classification systems are then used to guide risk assessment and case management to reduce offending risk and guide prevention (Knight & Prentky, 1990; Mandeville-Norden & Beech, 2009; Martínez-Catena et al., 2017; Robertiello & Terry, 2007; Ward & Siegert, 2002). Typologies also offer insight about motives driving CSA, clinical presentations, needs for intervention, etiological mechanisms, and offence trajectories. However, how well these offender-based typologies apply to persons who have sexual interest in children but never act on them is unclear. Rather than over-rely on risk-criminal based typologies, some researchers have called for typologies drawing from a clinical health-based perspective to better support individuals with these interests who are not justice-involved (Lievesley et al., 2020). Developing a typology of non-forensic MSICs in the community may clarify targets for interventions in such contexts to increase their psychological health, while also identifying avenues to prevent victimization of children that could be applied to the non-offending population.

## Risk Factors for Child Sexual Abuse

Multifactorial theories of CSA have indicated that a variety of developmental and biopsychosocial-sexual risk and protective factors function in dimensional, interactive, and mitigating ways to contribute to the perpetration of sexual offences against children, or lack-thereof (Hanson & Morton-Bourgon, 2005; Mann et al., 2010; Stephens et al., 2024; Ward & Beech, 2008). Integrating findings from theory and research, CSA risk factors rest across five biopsychosocial domains: 1) developmental factors (i.e., adverse childhood experiences, adult attachment style); 2) self-dysregulation (i.e., sexual preoccupation, impulsivity, affect regulation); 3) sexual interests (i.e., pedohebephilia, other paraphilic interests, sexual history); 4) distorted cognitions (i.e., pro-offending cognitions, emotional congruence with children); and 5) socio-affective deficits (i.e., mental health, distress, personality, loneliness).

Developmental risk factors reflecting adverse experiences during childhood and insecure attachment during adulthood can catalyze neurological and relational disturbances that may predispose men to other CSA risk factors. People with CSA offences have higher rates of sexual and/or physical victimization in childhood than people with sexual offences against adults and the general population (Bailey et al., 2016; Gerwinn et al., 2018; Levenson et al., 2016; Maniglio, 2011; Seto, 2018; Whitaker et al., 2008). People with CSA offences are more apt to have insecure attachment styles in relationships, which contributes to difficulties in interpersonal functioning and intimacy-related schemas (Gunst et al., 2017; Maniglio, 2011; Wood & Riggs, 2009).

Self-regulatory problems also are strong predictors of any recidivism among sexual offenders (Hanson & Morton-Bourgon, 2005). Inhibitory processing deficits may impair control of inappropriate sexual arousal, execution of sexual behaviours, and cognitive reappraisals of arousal to problematic stimuli (Mann et al., 2010). Compared to the general population, inhibitory control functions are significantly impaired among people who perpetrate CSA (Joyal et al., 2014). Impairments in neurobiological systems subsequently affect capacities for self-regulation within other sexual, psychosocial, cognitive, and behavioural systems that, in turn, perpetuate an individual’s risk for CSA.

Sexual deviancy, including attraction to children, has emerged as one of the strongest CSA risk factors (Hanson & Morton-Bourgon, 2005; Whitaker et al., 2008). The presence of pedohebephilic sexual interest is especially relevant as it offers additional pathways to interact with other biopsychosocial and situational risk factors to contribute to greater motivation and risk to perpetrate CSA. For example, exclusive pedophilic interests are associated with CSA perpetration and higher rates of sexual recidivism (Bailey et al., 2016). Additionally, people with multiple paraphilias (e.g., exhibitionism, voyeurism, sexual masochism, fetishism; APA, 2013) pose higher risk of sexual recidivism (Hanson & Morton-Bourgon, 2005).

Forensic literature recognizes antisocial cognitions as prominent risk factors for general offending, and for sexual offending specifically (Bonta & Andrews, 2024; Whitaker et al., 2008). Pro-offending cognitions foster beliefs that support or justify CSA (Ward & Keenan, 1999; Wood & Riggs, 2009). Likewise, experiencing higher emotional congruence with children, defined as “an exaggerated affective and cognitive affiliation with children and childhood… including emotional attachment and dependency needs … more likely met by interacting with children than with adults” (McPhail et al., 2013, p. 737), may interfere with forming healthy relationships with other adults or motivate desires for sexual and/or romantic experiences with children (Houtepen et al., 2016; Mann et al., 2010). The presence of both pedohebephilic sexual attraction and a wish to emotionally connect with children may contribute to increased sexual deviance and elevated risk of sexual behaviour with children (Konrad et al., 2018).

Socio-affective deficits increase vulnerability to dysregulation and criminal behaviours when one lacks capacity to adaptively manage their distress or navigate relationships (Hanson & Morton-Bourgon, 2005; Whitaker et al., 2008). Features related to mental health issues, personality traits, and loneliness are common among people who perpetrate CSA and may directly or indirectly have a role in the perpetration of CSA offences, as well as implications with barriers to help-seeking (e.g., stigma; Gunst et al., 2017; Hanson & Morton-Bourgon, 2005; Jahnke, Imhoff, et al., 2015; Jahnke, Schmidt, et al., 2015; Konrad et al., 2017; Mann et al., 2010; Stephens et al., 2024; Whitaker et al., 2008). In contrast, one’s ability to form intimate, supportive relationships with other adults can be protective against CSA (de Vries Robbé et al., 2015).

Despite the wealth of CSA criminal risk information, there is limited empirical evidence examining whether these same forensic population identified risk characteristics are relevant to “non-forensic” (i.e., no known sexual offending) MSICs living in the community. Applying these frameworks may still provide insight to understand heterogeneity among community MSICs as well as potential needs for preventative interventions. Since sexual interest in children is presumed to render men more at-risk for committing CSA, examining how community MSICs compare to groups of men with other, non-child oriented paraphilic interests can elucidate risk and protective factors when pedohebephilic interest is present.

## Current Study

There exists a subset of MSICs who have not, and some will not, sexually offend against children. Whereas some community MSICs report no issues with managing their sexual interests to refrain from CSA, others may be struggling to remain offence-free (Bailey et al., 2016). Thus, some MSICs may present elevated risk of committing CSA and some may experience significant distress and stigmatization regardless of offence status (Konrad et al., 2017). Research identifying differences between MSICs who do and do not sexually offend is in its infancy (Gerwinn et al., 2018). Extending MSIC research from forensic to non-forensic community samples may identify areas of need to inform preventative intervention against victimization. By expanding beyond risk-related priorities to include clinical health perspectives, development of services to focus on mental health and well-being of vulnerable persons may meet dual aims of personal wellness as well as by-product impacts toward prevention of risk and harm (Lievesley et al., 2020). Although paraphilic interest in general may elevate motivation for sexual offending, extant literature is clear in denoting etiological pathways to CSA that implicate increased vulnerability among some men with pedohebephilic interests. As such, the current research used a comparison group of men with other non-pedohebephilic paraphilic interests to examine how they compare on biopsychosocial-sexual factors with a heterogeneous group of non-forensic MSICs. Inclusion of such a comparison group also may facilitate identification of features that are transdiagnostic versus MSIC-specific targets for prevention efforts, as well as explore how characteristics may be unique to men who specifically identify a degree of sexual interest in children compared to men with paraphilias in general.

The primary goal of the current study was to explore the potential presence of clusters of community-based MSICs who report never criminally engaging in CSA. Men who endorsed sexual interest in children were recruited online to ensure anonymity and promote honest reporting. Extending theory and research on risk and protective factors for CSA, two major objectives were pursued. First, to identify underlying clusters of characteristics (e.g., developmental, self-regulatory, sexual, cognition, and socio-affective) among self-identified members of the non-criminal justice involved MSIC population to develop a psychometric-based typology. It was hypothesized that MSICs would be meaningfully grouped into at least two clusters reflecting meaningful variations in biopsychosocial-sexual characteristics. Second, to examine variations across biopsychosocial-sexual variables that best characterize and distinguish MSIC clusters. This included determining how profiles vary in risk/protective factors compared to MSICs with detected CSA and community men with other paraphilias, with variations expected in severity of clinical presentation.

## Method

### Participants and Sample Characteristics

Participants were screened based on inclusion criteria: 1) their sex/gender (i.e., male/men); 2) age category (≥18 years); 3) self-reported “non-traditional” or “atypical” (i.e., paraphilic) sexual interest of any type; 4) literacy in English reading/writing; 5) not having previously done the survey; and 6) informed consent to participate. Development of the typology only included men who reported sexual interests in children who have not been formally involved in the criminal justice system related to these interests (no reported charges or convictions for CSA offences). Men with other paraphilic interests and MSICs with historical CSA offences were used as comparison groups for Research Questions 2.

**Figure 1:**
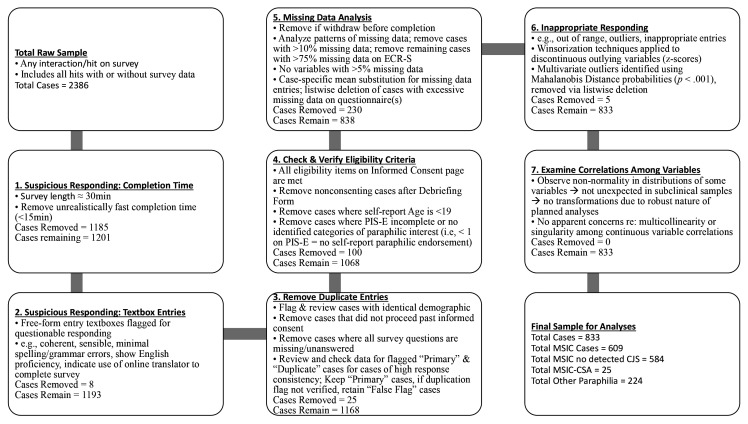
Summary of Data Cleaning and Conditioning Process From Initial Raw Data Sample to Final Sample Suitable for Statistical Analysis and Hypothesis Testing *Note.* Full description of process applied in this research is available in Stewart, 2022.

*A priori* power analysis required ≥132 participants to detect a medium effect size with adequate (i.e., 80%) statistical power with a .05 *p*-value for proposed analyses (Dziak et al., 2014; Tabachnick & Fidell, 2013). The present study aimed for a sample size of at least 250 MSICs to improve generalizability of results and account for unequal group sizes and oversampled (*N* ≈ 1,000) to ensure sufficient sample size for statistical power in the analyses (Dziak et al., 2014). A summary of steps taken for data cleaning and conditioning is described in Figure 1 (detailed overview available in Stewart, 2022). The final dataset contained 833 cases from the initial 2,386 with any survey interaction, and consisted of both MSIC participants (73.1%, *n* = 609; of whom 584 denied detected CSA offences) and other paraphilia participants (26.9%, *n* = 224).

#### Overall Sample Characteristics

MSIC and other paraphilia participants were recruited from online sources, including MSIC-related forums (50.6% and 2.2%, respectively), other social media forums (17.2% and 19.6%, respectively), MTurk (5.4% and 69.6%, respectively), or they did not disclose where they found out about the survey (26.8% and 8.5%, respectively).

On average, MSICs were 29.7 years old (*SD* = 9.81, range = 19 to 68) and mostly identified as Caucasian (79.8%) and residing in the United States (50.4%) or Europe (25.0%). Approximately two thirds (66.8%) were single in their relationship status. Most (91.3%) MSIC participants denied previous criminal history, with 4.1% (*n* = 25) reporting previous charge(s) for any sexual offence against a child below 16 years of age. These latter cases were allocated to the “MSIC-CSA” group. Thus, 584 MSIC participants who denied criminal justice involvement for CSA were included in typological hypotheses testing and analyses, whereas these other groups were used for comparison with the emerging clusters only.

Overall, 91.1% of all MSIC participants reported some degree of pedophilic arousal (i.e., child of any gender aged < 12 years) and 99.3% reported some degree of hebephilic arousal (i.e., child of any gender between ages of 12 to 14). Specifically, 90.0% of all MSICs endorsed arousal to girls aged ≤ 14 years, and 58.8% endorsed arousal to boys aged ≤ 14 years. Many MSICs also reported arousal to adult females (87.2%) and males (48.6%). Including pedophilia, hebephilia, and teleiophilia, MSICs typically endorsed some degree of sexual arousal to 7.26 (*SD* = 2.37, range = 2 to 13) categories of paraphilic interests. A minority of MSICs reported “exclusive” sexual interest in children (4.3%), whereas most MSICs reported non-exclusive sexual interest in children as well as adults (95.7%). Approximately one-quarter of the full sample denied any sexual arousal toward children (26.9%; *n* = 224); these participants were allocated to the Other Paraphilia group for comparative analyses.

Other Paraphilia, non-MSIC men had a mean age of 35.3 years (*SD* = 11.8, range = 19 to 77), were Caucasian (75.4%) and resided in the United States (81.7%) or Canada (11.2%). Relationship status was single for 41.5%. Most (91.1%) of Other Paraphilia group denied a criminal history, with 0.4% (*n* = 1) reporting previous charge(s) for a sexual offence against a child. Most reported paraphilic interests in Voyeurism (77.7%), Exhibitionism (69.2%), Fetishism (58.0%), Sexual Masochism (47.8%), and Frotteurism (43.8%). The Other Paraphilia group also endorsed some degree of sexual arousal to an average of 5.51 (*SD* = 2.27, range = 1 to 11) paraphilic categories.

### Procedure

The current study was approved by the Human Ethics Research Board of the authors’ affiliated institution. Online recruitment proceeded by posting recruitment advertisements across a variety of media including posts on social media, support groups and online forums, educative non-profit and prevention organizations, listservs for sex researchers and clinicians, and crowdsourcing research recruitment websites. The survey was hosted through SoSci Survey, a Germany-based online survey platform that administered and compiled data to ensure anonymity and confidentiality (Leiner, 2019). No identifiable participant data was collected. To foster honest participant engagement in a safe research environment (e.g., Klein et al., 2018; Ray et al., 2010), site moderators were contacted for permission to share the recruitment ad with their online communities.

After reviewing informed consent, eligible participants could voluntarily choose to complete the survey (~30 minutes in length). For questionnaires specific to cognitions (i.e., ABCS + CASA) or emotional congruence with children (CS-ECWC), participants were provided explicit definitions which stated that references to “child” or “children” referred to persons aged 14 years or younger, to align with definitional criteria used in this research. Participants recruited through MTurk (*n* = 300) were compensated $3.00 for their engagement as per expectations with that platform. Participants recruited more broadly across the Internet were offered entry into a draw to win one of ten $50 gift cards. Survey content was identical across both mediums.

### Materials

#### Demographic and Personal Characteristics

An author-constructed questionnaire collected information about participants’ age, relationship status, sexual orientation, ethnicity, country of residence, primary language, education, and employment. Self-reported information regarding criminal history also was collected. Regarding the latter, participants were asked to report if they “have ever been charged with a criminal offence, even if you received a pardon post-conviction” and if yes, to check off boxes of all “type(s)” of offences for which they have been charged. Regarding the “sexual offence” category, participants were further queried to specify “if yes, did this charge involve a sexual offence against a child (under 16 years old).”

#### Developmental Factors

##### Adverse Childhood Experiences

The Adverse Childhood Experiences-Revised (ACES-R; Finkelhor et al., 2015) measure uses 14-items to dichotomously assess exposure to adverse experiences during formative years. Total scores range from 0 to 14, with higher scores denoting exposure to a greater number of adverse childhood experiences. Exposure to four or more adverse childhood experiences is associated with higher risk of problems in areas of psychosocial and health functioning (i.e., clinically significant; Felitti et al., 1998). The original ACES has good internal consistency (α = .74; Karatekin & Hill, 2019) and good-to-excellent test-retest reliability (Dube et al., 2004). Finkelhor and colleagues (2015) demonstrated that the addition of the four contextual adversity items to the original 10-item scale led to more robust effects when measuring distress by trauma scores. The ACES-R internal reliability in the current study was acceptable for both the MSIC (α = .77) and Other Paraphilia (α = .84) groups.

##### Adult Attachment Style

The Experiences in Close Relationships – Short Form (ECR-S; Wei et al., 2007) is a 12-item, 7-point Likert scale assessing adult attachment styles in intimate relationships, wherein higher scores indicate higher levels of attachment insecurity. The ECR-S has assessed attachment patterns in close relationships in diverse research populations including undergraduate students (Wei et al., 2007), young adults, psychiatric patients with severe psychopathology, early adolescents, older adults, and individuals in same-sex relationships (Mikulincer & Shaver, 2016). The internal reliability for ECR-S attachment anxiety subscale and attachment avoidance subscale in the current study were all acceptable across MSIC (α = .78 and α = .79, respectively) and Other Paraphilia groups (α = .79 and α = .86, respectively).

#### Self-Regulatory Deficits

##### Sexual Preoccupation

The Hypersexual Behaviour Inventory (HBI; Reid et al., 2011) uses 19-items to assess proposed criteria for “hypersexual disorder” (Stewart & Fedoroff, 2014) using a 5-point Likert scale. Higher scores suggest higher sexual preoccupation and scores ≥ 53 are considered clinically elevated for men (Reid et al., 2011). Studies have found good internal consistency for each of the three subscales (Coping α = .86; Control α = .82; Consequences α = .75) and have supported the inventory’s structural validity (Bőthe et al., 2019; Reid et al., 2011). The internal reliability for the HBI-19 total score in the current study was excellent across MSIC and Other Paraphilia groups (α = .93 and α = .93, respectively).

##### Disinhibition and Impulsivity

The 20-item Short UPPS-P Impulsive Behaviour Scale (SUPPS-P; Lynam, 2013) uses a 4-point Likert scale to measure five facets of impulsivity. Higher subscale scores are indicative of higher levels of that facet of impulsivity. Cyders and colleagues (2014) found that the SUPPS-P demonstrated moderate-to-high construct validity in associations with subscales within the long form of the measure (UPPS-P). In the current study, the internal reliability for the SUPPS-P subscales was acceptable across MSIC and Other Paraphilia groups, including the negative urgency subscale (α = .74 and α = .82, respectively), lack of perseverance subscale (α = .67 and α = .77, respectively), lack of premeditation subscale (α = .80 and α = .83, respectively), sensation seeking subscale (α = .65 and α = .74, respectively), and positive urgency subscale (α = .80 and α = .85, respectively).

#### Deviant Sexual Interests

##### Sexual History

An author-constructed sexual history survey (SHS) consisted of five items to assess age at first: 1) masturbation; 2) viewing pornography; 3) any sexual contact with another person; and 4) consensual sexual intercourse. Respondents reported their total lifetime number of sexual partners. These data helped to descriptively contextualize subgroups.

##### Paraphilic Sexual Interests

The Paraphilic Interests Scale (Hsu et al., 2015) assessed self-reported sexual arousal to engaging in paraphilic behaviours described in the DSM-5 (APA, 2013). For the current study, additional items were included to assess arousal to chronophilia-related stimuli and up to three “other” self-specified sexual stimuli. Thus, the Paraphilic Interest Scale-Extended (i.e., PIS-E) as used in the present study comprised 20 items, with higher scores indicative of higher endorsement of paraphilic arousal. The PIS-E was used to allocate participants to “MSIC” versus “Other Paraphilia” designation. Specifically, men were allocated to the “MSIC” group when the participant reported a rating greater than 1 (i.e., “a little arousing” or higher) on any subscale item assessing arousal for “sexual activity involving a girl below the age of 12 years,” “sexual activity involving a boy below the age of 12 years,” “sexual activity involving a girl (age 12-14 years),” and/or “sexual activity involving a boy (age 12-14 years).” That is, men allocated to the Other Paraphilia group reported a score of 1 for all four of these items (i.e., “not arousing at all”). Total number of paraphilic categories was measured when rating on a subscale was greater than 1 (i.e., participant report the prompt as being “a little arousing” or higher). The total number of paraphilic categories endorsed was used for cluster analyses, whereas paraphilia subscale scores (excluding “other” category) allocated participants into either the MSIC or Other Paraphilia sample. In the current study, internal reliability for the PIS-E total score was acceptable across MSIC (α = .74) and Other Paraphilia groups (α = .70).

#### Offence-Supportive Cognitions

##### Pro-Offending Cognitions

Two measures were combined to assess cognitions about sexual activity with children. The 29-item Abel and Becker Cognition Scale (ABCS; Abel et al., 1984) is widely used in clinical and research settings to evaluate beliefs related to children and sex. Since items in the ABCS predominantly assess cognitive distortions related to sexual offending, the ABCS was extended with items from the Children and Sexual Activities Inventory (CASA; Howitt & Sheldon, 2007). The CASA was informed by Ward and Keenan’s (1999) five implicit theories to assess offence-supportive cognitions relevant to online sexual offenders (Howitt & Sheldon, 2007). Consistent with past research using this measure, the present study included ten additional items specific to child pornography offending (Howitt & Sheldon, 2007; Merdian, 2012; Merdian et al., 2014). Items 3, 4, 7, 9, 12, 13, 15, 16, 18 and 19 from the CASA were included in addition to the original ABCS items. Participants rated their agreement with 39 statements on a 5-point Likert scale. *Lower* scores show *higher* endorsement of CSA-supportive beliefs. In the current study, the internal reliability for the ABCS + CASA total score was good to excellent across MSIC (α = .96) and Other Paraphilia groups (α = .89).

##### Emotional Congruence With Children

The Emotional Congruence with Children Scale (CS-ECWC; Beckett, 1987) is a 15-item measure using a 5-point Likert scale to assess beliefs that adults can have “reciprocal”, emotionally satisfying relationships with children aged ≤12 years. The CS-ECWC has high internal consistency for the total score (α = .90; Beech, 1998; Fisher et al., 1999; Mandeville-Norden & Beech, 2009) and for individual factors (Positive Affect from Children α = .84; Special Relationships with Children α = .80; Preference for Relationships with Children α = .74; Waldron et al., 2006). Internal reliability for the CS-ECWC total score was excellent across MSIC (α = .92) and Other Paraphilia groups (α = .92).

#### Socio-Affective and Intimacy Deficits

##### Mental Health and Distress

The Depression, Anxiety, and Stress Scales-21 items (DASS-21; Lovibond & Lovibond, 1995) measures emotional states and symptoms associated with negative affect states. Higher scores indicate higher symptomatology. Cut-off scores above 60 denotes “severe” clinical severity (Beaufort et al., 2017). Recommended cut-offs for the Depression, Anxiety, and Stress subscales denote whether endorsed symptoms are considered normal (0-9, 0-7, 0-14, respectively), mild (10-13, 8-9, 15-18, respectively), moderate (14-20, 10-14, 19-25, respectively), severe (21-27, 15-19, 26-33, respectively), or extremely severe (28+, 20+, 34+, respectively; Lovibond & Lovibond, 1995). Evidence of excellent psychometric properties (i.e., reliability, construct validity, predictive validity) and clinical utility of the DASS-21 is supported across diverse general and clinical populations (Henry & Crawford, 2005; Norton, 2007; Parkitny & McAuley, 2010). Internal consistency for the DASS-21 total score was excellent across MSIC (α = .93) and Other Paraphilia groups (α = .95) in the current study.

##### Personality

The Personality Inventory for the DSM-5-Brief Form (PID-5-BF; Krueger et al., 2013) uses 25 self-report items on a 4-point Likert scale to measure dimensional conceptualizations of personality traits. Higher scores indicate more problematic personality traits. Subscales of the PID-5-BF itself have shown adequate internal consistency across each domain (Negative Affectivity α = .70; Disinhibition α = .76; Antagonism α = .68; Psychoticism α = .78; Detachment α = .69) in community and student samples (Anderson et al., 2018). In the current study, the internal reliability for the PID-5-BF total score was good to excellent across MSIC (α = .87) and Other Paraphilia groups (α = .91).

##### Loneliness

The 15-item Social and Emotional Loneliness Scale for Adults-Short Version (SELSA-S; DiTommaso et al., 2004) is distinct as it uses a multidimensional approach to measure loneliness. Since its development, the psychometric properties of the SELSA-S have been validated across diverse samples (e.g., internet users, university students, young adults, intimate partners, psychiatric outpatients; Adamczyk, 2016; DiTommaso et al., 2004, 2007; Ryan & Xenos, 2011). In the current study, the internal reliability for the SELSA-S subscales were good to excellent across MSIC and Other Paraphilia groups, including the romantic subscale (α = .85 and α = .86, respectively), family subscale (α = .88 and α = .91, respectively), and social subscale (α = .85 and α = .89, respectively).

### Data Analysis

Prior to conducting statistical analyses, data were screened and conditioned. An overview summarizing each step of data conditioning is summarized in Figure 1 (for detailed description of conditioning process, see Stewart, 2022).

Research Question 1 identified underlying *a posteriori* clusters from biopsychosocial-sexual factors of MSICs in the community who had no previous criminal justice system involvement for CSA (*n* = 584). Using MPlus 6.12, latent cluster analysis (LCA) identified latent subgroups of MSICs based on scores from measures of developmental (i.e., adverse experiences [ACES-R total score], adult attachment [ECR-S subscale scores]), self-regulatory (i.e., sexual preoccupation [HBI total score], impulsivity and disinhibition [SUPPS-P subscale scores]), sexual (i.e., paraphilic interests [PIS-E total number of paraphilic interest categories]), cognitive (i.e., pro-offending attitudes [ABCS + CASA total score], emotional congruence with children [CS-ECWC total score]), and socio-affective (i.e., mental health [DASS-21 total score], personality [PID-5-BF total score], loneliness [SELSA-S subscale scores]).

Using the latent groups from Research Question 1, Research Question 2 used descriptive analyses (i.e., Chi Square, frequencies, means, standard deviations) and MANOVA to examine which biopsychosocial-sexual factors significantly differ between MSIC clusters and men who endorse other paraphilic interests (i.e., Other Paraphilia; *n* = 225). The small subsample of community MSICs who self-reported prior charges or convictions for sexual offences against a child under the age of 16 years (i.e., MSIC-CSA; *n* = 25) were compared on mean scores across factors. Due to small sample size, analyses of MSIC-CSA group are a preliminary and exploratory effort to compare offender profiles with community MSICs with no detected CSA.

## Results

### Research Question 1: Develop MSIC Typology

LCA used 17 continuous measures of developmental, self-regulatory, sexual, cognitive, and socio-affective functioning to examine 2- to 10-class cluster solutions based on previous literature (i.e., forensic CSA typologies proposed by Beech, 1998; Groth et al., 1982; Henry et al., 2010; Knight & Prentky, 1990; Lanning, 1992; Mandeville-Norden & Beech, 2009; Ward & Siegert, 2002). The results of 2- to 6-class cluster solutions are summarized in Table 1. Maximum likelihood parameter estimates and observed scores were used to examine class membership probability for each *k*-class solution to minimize within-cluster variation, while also maximizing between cluster variation (Bakk & Vermunt, 2016; Vermunt & Magidson, 2002).

**Table 1:** Results of Latent Cluster Analysis of MSICs With no Previous Criminal Charges for Sexual Offences Against Children

Model	Participants % (*n*)	Maximum Loglikelihood	AIC	BIC	SSABIC	Entropy	Adjusted LMR (*p*)	BLRT (*p*)
2 class	56.8 (332)	-31426.1	62956.3	63183.5	63018.4	.853	1374.7 (< .001**)	-32119.5 (< .001**)
	43.2 (252)							
**3 class**	**28.3 (165)**	**-31233.0**	**62606.0**	**62911.9**	**62689.7**	**.825**	**382.9** (.036*)	**-31426.1** (< .001**)
	**46.2 (270)**							
	**25.5 (149)**							
4 class	26.4 (154)	-31124.4	62424.8	62809.3	62530.0	.813	215.4 (.255)	-31233.0 (< .001**)
	34.6 (202)							
	28.3 (165)							
	10.8 (63)							
5 class	14.4 (84)	-31023.6	62259.3	62722.5	62386.0	.823	199.8 (.212)	-31124.4 (< .001**)
	27.6 (161)							
	21.2 (124)							
	254.8 (145)							
	12.0 (70)							
6 class	11.5 (67)	-30935.2	62118.5	62660.4	62266.7	.860	175.3 (.108)	-31023.6 (< .001**)
	25.3 (148)							
	29.1 (170)							
	5.82 (34)							
	11.8 (69)							
	16.4 (96)							

*Note.* AIC = Akaike Information Criterion; BIC = Bayesian Information Criterion; SSABIC = sample-size adjusted BIC; LMR = Lo-Mendell-Rubin Likelihood Ratio Test; BLRT = Bootstrapped Likelihood Ratio Test. Bold text indicated the optimal class solution based on current study results.

**Figure 2:**
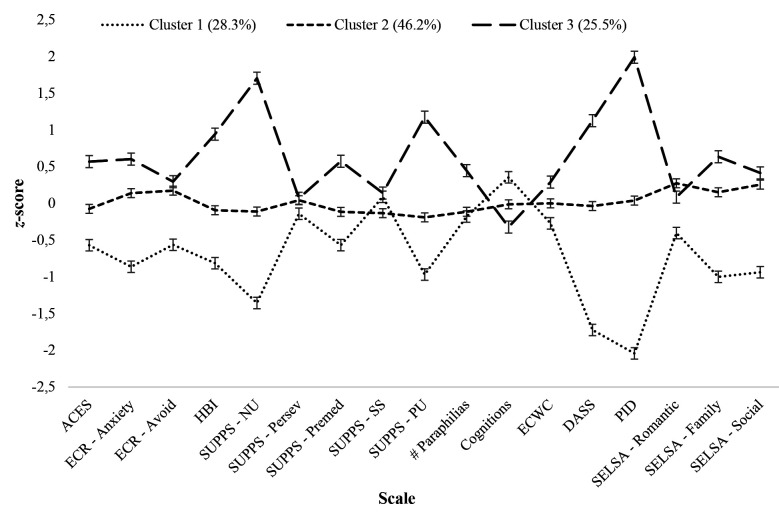
Standardized Mean Scores Depicting Latent Profiles of MSIC Participants With no Historical Charges for Sexual Offences Against Children Based on Developmental Factors, Self-Regulation Deficits, Deviant Sexual Interests, Offence-Supportive Cognitions, and Socio-Affective Factors

Based on recommended BIC, LMR, BLRT, entropy fit indices, and combined with nuanced interpretability and theoretical parsimony (Contractor et al., 2018; DiStefano & Kamphaus, 2006; Dziak et al., 2014; Nylund et al., 2007), a 3-class solution was selected as the optimal model for “undetected” community MSICs. Estimated membership probability for 3-class solution was 92.5% for Cluster 1, 95.0% for Cluster 2, and 90.4% for Cluster 3. Probability of misclassification was 7.5% for Cluster 1, 5.0% for Cluster 2, and 9.6% for Cluster 3. A graphical representation of z-score means for the 3-class solution is depicted in Figure 2.

Latent class membership differences for all measured characteristics included in the LCA are described in Table 2. Latent clusters were conceptually labelled according to significant patterns of biopsychosocial-sexual characteristics endorsed by members of those classes. Cluster labels were assigned based on major trends observed across characteristics and may not fully capture the nuances, heterogeneity, and complexity within clusters. Overall, the three clusters appear to represent different severity levels of vulnerability factors for community MSICs.

**Table 2:** Cluster Profiles: Comparison of Clusters on Biopsychosocial-Sexual Characteristics (n = 833)

Variable	Statistic			Cluster 1 “Low” *n* = 165	Cluster 2 “Moderate” *n* = 270	Cluster 3 “High” *n* = 149	MSIC – CSA *n* = 25	Other Paraphilia *n* = 224
	*F*	*p*	$\eta_{p}^{2}$	*M* (*SD*)	*M* (*SD)*	*M* (*SD*)	*M* (*SD*)	*M* (*SD*)
Developmental Factors	15.69	< .001	.102					
*ACES-R*				2.07 (2.30)	3.13 (2.51)	5.15 (3.11)	3.76 (2.62)	2.56 (2.97)
Abuse	21.52	< .001	.094	.38 (.68)^a^	.52 (.79)^a^	1.14 (1.11)^b^	1.04 (.89)^b^	.47 (.83)^a^
Neglect	17.02	< .001	.076	.12 (.36)^a^	.30 (.51)^ab^	.61 (.70)^c^	.36 (.64)^b^	.28 (.57)^ab^
Household Dysfunction	8.70	< .001	.040	.77 (.99)^a^	1.02 (1.12)^ab^	1.48 (1.34)^b^	1.00 (.82)^ab^	.88 (1.20)^a^
Contextual Adversity	28.97	< .001	.119	.81 (1.04)^a^	1.36 (1.10)^b^	1.93 (1.06)^c^	1.36 (1.15)^b^	.93 (1.03)^ab^
*ECR-S*								
Attachment Anxiety	58.57	< .001	.221	18.65 (6.04)^a^	24.74 (6.29)^c^	28.04 (6.93)^d^	22.08 (8.47)^bc^	19.18 (7.60)^ab^
Attachment Avoidance	38.96	< .001	.158	16.68 (6.37)^a^	21.43 (6.62)^b^	22.19 (6.46)^b^	20.61 (8.12)^b^	15.50 (6.67)^a^
Self-Regulation Factors	16.35	< .001	.137					
*HBI-19*				36.37 (12.53)	45.31 (13.69)	60.20 (14.45)	51.49 (18.99)	40.78 (13.49)
Control	60.04	< .001	.225	15.15 (6.89)^a^	19.40 (8.01)^b^	26.17 (7.26)^c^	23.25 (9.39)^c^	15.81 (8.29)^a^
Coping	34.64	< .001	.143	15.11 (6.57)^a^	18.15 (6.71)^b^	23.81 (7.11)^c^	19.20 (8.23)^b^	17.97 (6.27)^b^
Consequences	38.52	< .001	.157	6.11 (2.82)^a^	7.76 (3.09)^bc^	10.22 (3.66)^d^	9.04 (4.31)^c^	7.00 (2.89)^ab^
*SUPPS-P*								
Negative Urgency	128.01	< .001	.382	6.68 (1.91)^a^	9.04 (2.08)^bc^	12.56 (1.93)^d^	9.43 (3.43)^c^	8.41 (3.01)^b^
Lack of Perseverance	6.83	< .001	.032	7.62 (2.06)^ab^	8.00 (2.07)^ab^	8.07 (2.43)^b^	7.99 (2.28)^ab^	7.08 (2.32)^a^
Lack of Premeditation	26.66	< .001	.114	5.90 (1.62)^a^	6.58 (2.08)^ab^	8.26 (2.51)^c^	7.36 (2.41)^bc^	6.53 (2.23)^ab^
Sensation Seeking	2.27	.061	.011	9.92 (2.78)^a^	9.29 (2.86)^a^	10.08 (2.91)^a^	9.68 (2.67)^a^	9.85 (3.25)^a^
Positive Urgency	76.39	< .001	.270	5.64 (1.92)^a^	7.08 (2.21)^b^	10.33 (2.41)^c^	7.64 (3.21)^b^	7.36 (2.92)^b^
Cognitive Factors	15.71	< .001	.147					
*ABCS + CASA*				153.65 (26.30)	143.98 (27.07)	134.56 (30.29)	154.36 (31.30)	184.8 (10.5)
Sexual Objectification of Children	124.33	< .001	.375	48.78 (11.43)^bc^	45.61 (11.15)^ab^	42.69 (11.99)^a^	50.50 (12.85)^c^	62.78 (3.54)^d^
Justification	55.69	< .001	.212	21.95 (3.40)^b^	20.87 (4.00)^ab^	19.45 (4.71)^a^	21.84 (3.68)^b^	24.53 (1.19)^d^
Children as Sexual Agents	64.50	< .001	.238	21.73 (3.39)^b^	20.49 (3.72)^ab^	19.15 (4.13)^a^	21.12 (4.57)^b^	24.23 (1.47)^d^
Denial of Sexual Offender Status^†^	65.03	< .001	.239	24.97 (4.47)^b^	22.34 (4.55)^ab^	20.61 (4.91)^a^	21.88 (4.66)^a^	27.24 (3.28)^c^
Emphasis on Cognitive Elements	137.82	< .001	.400	13.92 (2.84)^b^	12.98 (2.87)^ab^	12.49 (3.08)^a^	15.33 (2.99)^c^	18.05 (2.05)^d^
Power and Entitlement	93.02	< .001	.310	20.54 (3.56)^b^	19.07 (3.84)^a^	17.87 (4.38)^a^	20.84 (3.98)^b^	24.03 (1.44)^c^
*CS-ECWC*				20.78 (14.41)	24.71 (15.85)	29.04 (14.95)	25.44 (18.21)	10.19 (11.24)
Positive Affect from Children	40.56	< .001	.164	11.62 (8.14)^b^	13.39 (9.01)^bc^	15.79 (8.55)^c^	14.40 (8.97)^bc^	5.90 (6.85)^a^
Special Relationship with Children	36.83	< .001	.151	6.91 (5.12)^b^	8.19 (5.74)^bc^	9.84 (5.45)^c^	7.68 (7.28)^bc^	3.63 (4.36)^a^
Preference for Child Relationships	38.45	< .001	.157	2.25 (2.51)^b^	3.13 (2.80)^bc^	3.41 (2.91)^c^	3.36 (2.93)^c^	3.36 (2.93)^a^
Sexual Factors	9.06	< .001	.090					
*PIS-E*								
Total # Paraphilia Categories	34.56	< .001	.143	6.87 (2.46)^b^	7.05 (2.28)^b^	8.27 (2.15)^c^	6.12 (2.20)^ab^	5.51 (2.27)^a^
MSIC Attraction (≤ 14 years)	1.32	.267	.007	2.85 (.97)^a^	3.03 (.90)^a^	2.97 (.89)^a^	3.06 (.98)^a^	-
Female	1.70	.167	.008	3.35 (1.41)^a^	3.64 (1.29)^a^	3.51 (1.37)^a^	3.32 (1.53)^a^	-
Male	.65	.581	.003	2.35 (1.54)^a^	2.42 (1.53)^a^	2.43 (1.39)^a^	2.80 (1.51)^a^	-
Teleiophilia	9.93	< .001	.046	2.89 (.84)^b^	2.72 (2.72)^ab^	2.89 (.96)^b^	2.52 (1.08)^a^	3.16 (.72)^c^
Pedophilia	2.73	.043	.013	2.71 (1.14)^a^	3.01 (1.12)^a^	2.95 (1.06)^a^	3.02 (1.09)^a^	-
Hebephilia	.23	.878	.001	3.00 (.92)^a^	3.04 (.83)^a^	2.99 (.91)^a^	3.10 (1.05)^a^	-
Exhibitionism	7.23	< .001	.034	1.89 (1.10)^a^	1.81 (.99)^a^	2.33 (1.19)^b^	1.62 (.85)^a^	1.96 (.97)^ab^
Voyeurism	4.65	.001	.022	2.17 (1.13)^ab^	2.16 (1.06)^ab^	2.59 (1.16)^b^	2.02 (1.26)^a^	2.37 (1.17)^ab^
Sexual Sadism	7.70	< .001	.036	1.48 (.83)^a^	1.54 (.91)^a^	2.00 (1.25)^b^	1.44 (.94)^a^	1.54 (.91)^a^
Sexual Masochism	11.74	< .001	.054	1.37 (.79)^ab^	1.47 (.84)^ab^	1.98 (1.15)^c^	1.24 (.69)^a^	1.67 (.91)^bc^
Fetishism	4.09	.003	.019	1.89 (1.15)^a^	1.98 (1.12)^a^	2.28 (1.18)^a^	1.80 (1.08)^a^	2.25 (1.33)^a^
Transvestism	5.13	< .001	.024	1.72 (1.15)^a^	1.83 (1.21)^ab^	2.25 (1.31)^b^	1.80 (1.29)^ab^	1.73 (1.18)^a^
Frotteurism	7.11	< .001	.033	1.67 (1.67)^a^	1.80 (1.06)^ab^	2.24 (1.30)^b^	1.52 (.87)^a^	1.75 (1.05)^a^
Socio-affective Factors	17.86	< .001	.193					
*DASS-21*				16.34 (12.68)	37.54 (18.05)	63.55 (22.51)	38.20 (25.97)	25.53 (23.05)
Stress	95.12	< .001	.315	5.84 (5.47)^a^	11.01 (6.90)^bc^	21.44 (8.17)^d^	13.92 (9.70)^c^	9.75 (8.37)^b^
Anxiety	72.69	< .001	.260	3.33 (4.02)^a^	8.16 (6.46)^b^	16.12 (9.09)^c^	8.48 (7.60)^b^	5.90 (7.81)^ab^
Depression	95.91	< .001	.317	7.17 (6.81)^a^	18.37 (10.16)^b^	25.99 (10.68)^c^	15.80 (13.3)^b^	9.88 (10.24)^a^
*PID-5-BF*				14.16 (6.41)	27.51 (6.75)	41.26 (7.04)	24.76 (13.45)	19.49 (12.89)
Negative Affect	106.64	< .001	.340	.69 (.51)^a^	1.29 (.51)^b^	1.92 (.51)^c^	1.20 (.74)^b^	.91 (.70)^a^
Detachment	62.39	< .001	.232	.82 (.65)^a^	1.58 (.74)^bc^	1.77 (.74)^c^	1.42 (.68)^b^	.92 (.73)^a^
Antagonism	38.94	< .001	.158	.40 (.42)^a^	.68 (.55)^b^	1.15 (.64)^c^	.64 (.66)^b^	.59 (.57)^ab^
Disinhibition	100.87	< .001	.328	.34 (.37)^a^	.76 (.54)^bc^	1.60 (.68)^d^	.95 (.75)^c^	.66 (.66)^b^
Psychoticism	100.30	< .001	.326	.62 (.52)^a^	1.24 (.54)^b^	1.85 (.58)^c^	.81 (.70)^a^	.84 (.72)^a^
*SELSA-S*								
Romantic	39.0	< .001	.160	20.48 (10.55)^a^	276.95 (8.07)^b^	25.46 (8.39)^b^	24.40 (9.53)^b^	17.15 (9.36)^a^
Family	59.03	< .001	.222	10.69 (6.12)^a^	17.91 (7.24)^b^	21.60 (7.54)^c^	17.06 (8.10)^b^	13.20 (7.37)^a^
Social	43.79	< .001	.175	12.35 (6.37)^a^	20.28 (7.67)^b^	21.38 (7.37)^b^	19.08 (8.72)^b^	15.43 (7.81)^a^

*Note.* Groups that share superscripts are not significantly different from one another in *post-hoc* analysis (Tukey’s HSD Test; *p* < .05). ^†^Subscale label from Merdian et al. (2014), where component captured themes of uncontrollability of CSA situations and beliefs of being able to minimize harm to victims (i.e., denial of harm/controllability, versus denial of CSA criminal offence perpetration).

Participants classified in Cluster 1 (*n* = 165, 28.3%) were characterized by lower levels of developmental and biopsychosocial-sexual vulnerability factors, thus labelled *Low Vulnerability*. Members of Cluster 1 endorsed significantly lower adverse childhood experiences, attachment anxiety, attachment avoidance, sexual preoccupation, negative urgency, lack of premeditation, positive urgency, pro-offending attitudes, emotional congruence with children, mental health disturbance, mildly problematic personality traits, romantic loneliness, familial loneliness, and social loneliness compared to participants in other clusters. Participants in Cluster 1 also had significantly lower scores on number of paraphilic interests compared to men in Cluster 3.

MSICs classified in Cluster 2 (*n* = 270, 46.2%) demonstrated intermediate endorsement of all measured vulnerability factors and labelled *Moderate Vulnerability*. Cluster 2 MSICs showed higher endorsement than Cluster 1 and lower endorsement than Cluster 3 on measures of adverse childhood experiences, attachment anxiety, sexual preoccupation, negative urgency, lack of premeditation, positive urgency, pro-offending attitudes, emotional congruence with children, mental health disturbance, problematic personality traits, family loneliness. Men in Cluster 2 also had significantly lower scores on number of paraphilic interests compared to Cluster 3 and significantly higher scores of romantic loneliness and social loneliness compared to Cluster 1.

Finally, Cluster 3 (*n* = 149, 25.5%) MSICs were distinguished by higher endorsement of most biopsychosocial-sexual vulnerability factors and was named *High Vulnerability*. Of note, average endorsement of adverse childhood experiences, sexual preoccupation, and mental health disturbance were above recommended cut-offs of clinical severity for participants in Cluster 3.

Compared to participants in Cluster 1 and Cluster 2, MSICs in Cluster 3 endorsed significantly higher adverse childhood experiences (clinically significant; see Materials section), attachment anxiety, sexual preoccupation (clinically significant; see Materials section), negative urgency, lack of premeditation, positive urgency, pro-offending attitudes, emotional congruence with children, mental health disturbance (clinically significant; see Materials section), moderately high problematic personality traits, and family loneliness. Higher attachment avoidance, sensation seeking, total number of paraphilic interests, romantic loneliness, and social loneliness also were observed for participants in Cluster 3 compared to those in Cluster 1.

### Research Question 2: Compare and Contrast Group Characteristics

Descriptive analyses of individual characteristics explored differences between the MSIC subtypes and Other Paraphilia groups. Results are summarized in Table 3. Although dispersed internationally, most participants across all groups were similar in being white North American men who were educated and employed. On average, they ranged in age from their late-twenties to early/mid-thirties. The full sample showed little propensity for antisocial behaviour as per self-reported criminal history. For sexual history, MSIC groups tended to report younger ages of onset but concurrently were less sexually experienced than Other Paraphilia group. This finding aligns with forensic literature suggesting that people with pedohebephilic interests or histories of CSA offences begin sexual behaviours at younger ages (e.g., Gerwinn et al., 2018; Houtepen et al., 2016; Levenson et al., 2017). Among Moderate Vulnerability MSICs, limited sexual and relationship history suggests challenges with intimacy and social aptitude.

**Table 3:** Cluster Profiles: Comparison of Clusters on Demographic and Individual Characteristics (n = 808)

Variable	Statistic			Cluster 1 “Low” *n* = 165	Cluster 2 “Moderate” *n* = 270	Cluster 3 “High” *n* = 149	Other Paraphilia *n* = 224
Demographic Characteristic	*F*	*p*	$\eta_{p}^{2}$	*M* (*SD*)	*M* (*SD*)	*M* (*SD*)	*M* (*SD*)
Age	24.9	< .001	.124	31.3 (9.67)^ab^	28.0 (8.79)^a^	28.4 (8.26)^a^	35.3 (11.8)^b^
Total Criminal History	63.8	< .001	.236	.042 (.230)^a^	.067 (.304)^a^	.101 (.415)^a^	.134 (.492)^a^
Demographic Characteristic	χ^2^	*p*	Cramer’s *V*	% (*n*)	% (*n*)	% (*n*)	% (*n*)
Relationship Status	69.20	< .001	.207				
Single				55.8 (92)	75.6 (204)	67.1 (100)	41.7 (93)
In a Relationship/Married				41.8 (69)	20.0 (54)	28.2 (42)	53.4 (119)
Other				2.4 (4)	4.4 (12)	4.7 (7)	4.2 (34)
Sexual Orientation	31.43	< .001	.139				
Heterosexual				54.5 (90)	47.0 (127)	39.6 (59)	66.1 (148)
Homosexual				10.9 (18)	9.6 (26)	12.1 (18)	8.0 (18)
Other				34.5 (57)	43.3 (117)	48.3 (72)	25.9 (58)
Race/Ethnicity	5.10	.164	.080				
Caucasian				82.9 (136)	81.0 (217)	75.2 (112)	75.4 (169)
Other				17.1 (28)	19.0 (51)	24.8 (37)	24.6 (55)
Country of Residence	97.48	< .001	.248				
Canada / USA				59.2 (97)	57.3(152)	64.3(92)	93.3(208)
Other				40.9 (67)	42.6 (113)	35.7 (51)	6.7 (15)
Primary Language	48.41	< .001	.246				
English				70.9 (117)	70.4 (188)	77.7 (115)	94.2 (210)
Other				29.1 (48)	29.6 (79)	22.3 (33)	5.8 (13)
Education Status	23.62	< .001	.171				
High School/GED or Less				10.9 (18)	23.0 (62)	28.2 (42)	12.9 (29)
More than High School				89.1 (147)	77.0 (207)	71.8 (107)	87.1 (195)
Employment Status	57.91	< .001	.155				
Student				18.8 (31)	20.0 (54)	21.5 (32)	11.2 (25)
Employed				67.3 (111)	55.2 (149)	43.6 (65)	76.3 (171)
Unemployed				11.5 (19)	21.9 (59)	32.2 (48)	9.4 (21)
Other				2.4 (4)	3.0 (8)	2.7 (4)	3.1 (7)
Any Criminal History	6.22	.101	.088	3.0 (5)	5.6 (15)	5.4 (8)	8.9 (20)
Age First Masturbation	81.54	< .001	.227				
< 13 years old				82.4 (131)	79.6 (211)	80.7 (117)	52.5 (116)
13-19 years old				17.6 (28)	20.0 (53)	16.6 (24)	47.5 (105)
20+ years old				0.0 (0)	.4 (1)	2.8 (4)	0.0 (0)
Age First Pornography	51.61	< .001	.180				
< 13 years old				73.5 (119)	76.5 (202)	78.8 (115)	55.2 (122)
13-19 years old				25.9 (42)	22.7 (60)	17.1 (25)	44.3 (98)
20+ years old				.6 (1)	.8 (2)	4.1 (6)	.5 (1)
Age First Sexual Contact	95.91	< .001	.200				
None				18.4 (30)	28.3 (75)	15.0 (22)	5.4 (12)
< 13 years old				23.9 (39)	29.8 (79)	33.3 (49)	13.1 (29)
13-19 years old				46.0 (75)	34.0 (90)	43.5 (64)	72.1 (160)
20+ years old				11.7 (19)	7.9 (21)	8.2 (12)	9.5 (21)
Age First Consensual Intercourse	117.65	< .001	.222				
None/Never				29.4 (48)	43.0 (113)	25.2 (37)	5.9 (13)
< 13 years old				8.6 (14)	6.5 (17)	8.8 (13)	3.2 (7)
13-19 years old				42.3 (69)	34.2 (90)	44.9 (66)	76.0 (168)
20+ years old				19.6 (32)	16.3 (43)	21.1 (31)	14.9 (33)
Lifetime Sexual Partners	125.95	< .001	.230				
0/None				27.2 (44)	41.1 (108)	24.8 (36)	5.4 (12)
1-5				46.2 (69)	41.8 (110)	49.0 (71)	39.8 (88)
6-10				11.1 (18)	11.0 (29)	13.8 (20)	22.2 (49)
10+				19.1 (31)	6.1 (16)	12.4 (18)	32.6 (72)

*Note*. Groups that share superscripts are not significantly different from one another using Tukey’s HSD Test *for post-hoc* analysis (*p* < .05).

Moderate and High Vulnerability groups had relatively lower academic achievement and higher unemployment than the Low Vulnerability and Other Paraphilia groups. The MSIC groups more often identified heterodivergent orientation and reported being single, suggesting difficulties with adult intimate relationships particularly among the Moderate and High Vulnerability groups.

Next, five multivariate analysis of variance (MANOVA) examined which variables best differentiated between the identified MSIC groups, MSIC-CSA men, and men with other paraphilias (Cramer, 2003; Mertler & Vannatta, 2002; Tabachnick & Fidell, 2013). MANOVAs used participants’ self-reported scores on measures’ subscales as dependent variables to identify groups differences. Follow-up univariate ANOVA and Tukey’s post hoc analyses (*p*-value ≤ .05) are summarized for all MANOVAs in Table 2.

#### Developmental Factors

The multivariate effect was significant for developmental factors, *Pillai’s Trace* = .409, *F*(24, 3304) = 15.7, *p* < .001, $\eta_{p}^{2}$ = .102, indicating a moderately large effect size of group difference in endorsement of developmental factors (see Table 2). For adverse childhood experiences, follow-up ANOVAs found significant differences between groups for experiences of abuse, neglect, household dysfunction, and contextual adversity. Follow-up ANOVAs saw significant differences between groups on anxiety and avoidance facets of adult attachment. Overall, High Vulnerability MSICs endorsed the highest occurrence of all different types of adverse childhood experiences. Furthermore, High Vulnerability MSICs have more of an insecure attachment style relative to other groups, with similar levels of attachment avoidance as the Moderate Vulnerability and MSIC-CSA groups. In contrast, the Low Vulnerability and Other Paraphilia groups endorsed more secure attachment styles.

#### Self-Regulatory Factors

The multivariate effect was significant for self-regulatory factors, *Pillai’s Trace* = .548, *F*(32, 3296) = 16.4, *p* < .001, $\eta_{p}^{2}$ = .137, denoting a moderately large effect size of group difference in endorsement of self-regulatory factors (see Table 2). For sexual preoccupation, follow-up ANOVAs showed significant differences between groups for sexual control, sexual coping, and sexual consequences. Follow-up ANOVAs saw significant differences across groups for all impulsivity facets except for sensation seeking. The High Vulnerability group reported hypersexuality surpassing clinical thresholds and are prone to act rashly in response to any strong affective experience. Levels of hypersexuality and impulsivity among the Moderate Vulnerability and MSIC-CSA groups were similar. The Low Vulnerability group had lowest propensity for disinhibition.

#### Cognitive Factors

Regarding pro-offending cognitions, the reader is advised to note that lower scores indicate higher endorsement of cognitive distortions on the ABCS+CASA. The multivariate effect was significant for cognitive factors, *Pillai’s Trace* = .586, *F*(36, 3292) = 15.7, *p* < .001, $\eta_{p}^{2}$ = .147, indicating a large effect size of group difference in support of cognitive factors (see Table 2). In terms of distorted beliefs about children and sex, follow-up ANOVAs found significant differences between groups regarding sexual objectification of children, justification, children as sexual agents, denial of sexual offender status, emphasis on cognitive elements, and power and entitlement. Follow-up ANOVAs also observed significant differences between groups for emotional congruence with children, including beliefs about deriving positive affect from children, perceptions about having special relationships with children, and attitudes showing preference for relationships with children. Whereas most MSIC subgroups tended to disagree CSA-supportive attitudes, the High Vulnerability group trended toward neutrality. Regarding emotional congruence with children, the Moderate Vulnerability, High Vulnerability, and MSIC-CSA groups were more ambivalent than the Low Vulnerability group. The Other Paraphilia group considered these beliefs to be very untrue.

#### Sexual Interest Factors

The multivariate effect was significant for sexual interest factors, *Pillai’s Trace* = .361, *F*(36, 3292) = 9.06, *p* < .001, $\eta_{p}^{2}$ = .09, indicating a moderate effect size of group difference in endorsement of sexual factors (see Table 2). Given that arousal to pedophilic and/or hebephilic behaviours was used to differentiate between men with any degree of sexual interest in children from men with other paraphilic interests, a separate MANOVA was performed to examine differences in arousal to children exclusively among MSIC groups. The multivariate effect was not significant for factors related to sexual attraction to children, Pillai’s Trace = .026, *F*(9, 1812) = 16.4, *p* < .067, $\eta_{p}^{2}$ = .009. Follow-up ANOVAs showed significant differences across groups in their endorsed sexual arousal to behaviours indicative of teleiophilia, exhibitionism, voyeurism, sexual sadism, sexual masochism, fetishism, transvestism, frotteurism, and total number of endorsed paraphilic categories. In sum, the High Vulnerability group endorsed highest paraphilic arousal and versatility. All MSIC groups reported similar levels of arousal to pedophilic and hebephilic stimuli, typically indicating that these stimuli were “somewhat” arousing.

#### Socio-Affective Factors

The multivariate effect was significant for socio-affective factors, *Pillai’s Trace* = .772, *F*(44, 3284) = 17.9, *p* < .001, $\eta_{p}^{2}$ = .193, indicating a large effect size of difference in socio-affective factors between groups (see Table 2). Follow-up ANOVAs observed significant differences between groups in their self-reported experiences of mental health-related distress over the past week, including symptoms of stress, anxiety, and depression. In follow-up ANOVAs, personality profiles across groups were significantly different for personality facets of negative affectivity, detachment, antagonism, disinhibition, and psychoticism. For experiences of loneliness, follow-up ANOVAs found significant differences between groups in terms of romantic loneliness, family loneliness, and social loneliness.

## Discussion

### Summarizing Profiles of MSIC Clusters in the Vulnerability Typology

The emergent Vulnerability Typology revealed that clusters of men endorsing sexual interest in children with no detected CSA histories were primarily distinguished based on self-reported intensity of biopsychosocial-sexual risk factors associated with perpetration of CSA. The Low Vulnerability MSIC cluster (28.2%) reported normal, unimpaired levels of measured vulnerability characteristics. This profile demonstrated more social stability and aptitude, better capacity for self-regulation, and lower developmental predisposition to risk factors despite their sexual interest in children. These strengths may offer protective effects against perpetrating CSA. In contrast, the largest proportion of MSICs were classified in the Moderate Vulnerability cluster (46.2%). Overall, this second profile was characterized by intermediate impairment across domains, including indications of elevated sociosexual challenges. Although the Moderate Vulnerability cluster may not be as “globally” distressed as the High Vulnerability group, they may not derive benefit from protective factors to the same degree as the Low Vulnerability group. The High Vulnerability cluster (25.5%) of the MSICs group and was characterized by global functional deficits across all biopsychosocial domains of interest, which is indicative of broad clinical and criminogenic needs relevant to risk factors associated with CSA. The presence of numerous dysfunctional mechanisms among High Vulnerability MSICs may render them more susceptible to multiple etiological pathways leading to sexual offending. Recent literature examining MSIC populations with detected and undetected CSA histories found that those with CSA histories tend to score highest on measured biopsychosocial-sexual factors, suggesting that the High Vulnerability group may especially benefit from interventions to prevent perpetration of CSA (Stephens et al., 2024).

### Linking the Vulnerability Typology to Forensic Literature

Aligning with existing forensic typologies and theoretical models, vulnerability emerges as a multifaceted phenomenon, involving proximal and distal factors that may increase risk for problematic functional outcomes, whether legal or otherwise (e.g., Hanson & Morton-Bourgon, 2005; Mann et al., 2010). The dynamic nature of identified vulnerability factors offers intervention targets to reduce risk for criminal behaviour, increase wellness, and build good lives. Whereas external or internal stressors may destabilize and increase one’s vulnerability to problematic consequences, it is possible that well-matched, proactive intervention informed by evidence-based clinical models may shift MSICs to lower vulnerability status. By extending the forensic literature, the Vulnerability Typology provides a framework to guide application and prioritization of therapeutic and risk management targets for community MSICs.

Congruent with the Risk-Need-Responsivity (RNR) model of criminal behaviour and crime prevention (Bonta & Andrews, 2024), the Vulnerability Typology suggests merit in having service intensity graded to match the level of criminogenic and clinical need reflected in different MSIC profiles. Overall, results of the current study suggest that the needs of Low Vulnerability profiles may best be addressed with low intensity intervention, or may not require any intervention if their protective features are sufficient to support ongoing prosocial self-management. Moderate-intensity intervention would be suitable for MSICs with Moderate Vulnerability profiles, adopting a skill-building approach to increase effective strategies for managing deviant sexual interests and navigating life stressors. Finally, the globally elevated level of need characterizing High Vulnerability profiles represents multiple areas to target in intervention, which can warrant longer-term, higher-intensity treatment efforts.

### Clinical Considerations for Prevention Approaches

Overall, the current findings show that men’s vulnerability for CSA perpetration is not necessarily contingent on a single problematic risk factor (e.g., pedohebephilia), but more likely emerges from the combined effects of several co-occurring risk and destabilizing factors. Facets of psychosocial wellness may exert destabilizing effects that elevate susceptibility to other criminogenic factors, potentially moving closer to crossing legal sexual lines in parallel with heightened subjective distress. Establishing secondary prevention programs may improve accessibility of therapeutic resources for MSICs to support them in remaining offence-free while concurrently addressing a variety of general mental health issues and criminogenic needs affecting their daily functioning and risk potential (Beier et al., 2009; Lasher & Stinson, 2017; Stephens & McPhail, 2019). The current study supports the notion that, regardless of their offence status, community MSICs *do* report challenges across an array of dynamic factors, many of which may be intervention targets. These findings further imply that help-seeking MSICs may not necessarily present at clinical service agencies for concerns explicitly related to their sexual interests, but nevertheless may benefit from risk-informed intervention services. For example, MSICs in the community may present to treatment for concerns such as coping with internalized stigma or general mental health, in addition to seeking help for their pedohebephilic interests (Stephens et al., 2024). In combination with research showing high rates of mental health disorders and related concerns (e.g., suicidality, substance misuse, internalized stigma) among community MSICs (Elchuk et al., 2022; McPhail & Olver, 2020; Stephens & McPhail, 2019; Stephens et al., 2024), this research highlights the benefit of addressing specific mental health and intimacy needs among this population. CSA Efforts to prevent CSA would best serve at-risk MSICs by addressing both criminogenic and possibly destabilizing psychological needs.

Although not explicitly assessed in the current study, it is likely that shame and stigma influence subjective experiences and present additional psychological need targets to address in prevention and intervention efforts (Elchuk et al., 2022; Jahnke, Imhoff, et al., 2015; Jahnke, Schmidt, et al., 2015). Indeed, associations exist between internalized stigma, loneliness, and psychological distress among populations with sexual interest in children (Elchuk et al., 2022; Stephens et al., 2024). Integration of principles prescribed by RNR and Good Lives Model (Ward & Gannon, 2006; Ward & Laws, 2010; Ward & Marshall, 2004; Ward & Stewart, 2003), in addition to practices adopted in humanistic, person-centered, and trauma-informed orientations (Levenson & Grady, 2019; Lievesley et al., 2020; Walker, 2021), may help match intervention intensity to equip MSICs with internal and external resources to develop capacity, skills, and resources to attain goals in non-offending ways to live a better life. Preventative intervention with MSICs can balance dual aims of personal wellness and public safety by promoting stability (i.e., management of impulses and emotions), accountability (i.e., safe decision making, supportive prosocial support network), and plans (i.e., engagement in fulfilling activities and prosocial goals) toward a life that is socially acceptable, personally meaningful, and subjectively worth living. Some preventative intervention efforts that have emerged to address the dual foci of targeting criminogenic and destabilizing psychological needs among MSICs have included Germany’s Project Dunkelfeld, Canada’s Talking for Change, and online peer-support forums such as Virtuous Pedophiles, B4U-ACT, and HelpWanted.

### Strengths and Limitations

A strength of the current study was the substantial effort made to maximize recruitment via safeguards to assure participant anonymity, confidentiality, and honest responding among a stigmatized and hard-to-reach population (Klein et al., 2018; Ray et al., 2010). We strove to maintain transparency by communicating directly with group representatives and moderators for permission to share the study with community members, as well as recruiting participants from other broader online mediums. These approaches resulted in a sufficiently large sample to investigate research hypotheses as proposed. In addition, we were comprehensive in assessing many constructs relevant to understanding biopsychosocial-sexual MSIC profiles using psychometrically appropriate measures.

Notwithstanding the strengths of the current study, results must be interpreted considering several limitations. Most significantly, self-report questionnaires present potential for bias in subjective self-evaluations. In addition, online data collection from a hidden population presents benefits and pitfalls. Online research methodologies can encourage self-disclosure and increase reach to diverse participant pools but presents challenges in verifying participant eligibility and self-selection bias. Thus, participants completed pre-screening items as part of the informed consent to access the survey. Low-quality data were removed during conditioning processes. Questionnaires were disseminated across a variety of websites to reduce sampling bias, but cultural norms (e.g., mandatory reporting, ease of accessibility), forums’ philosophical attitudes, and perceived acceptability of the research’s rationale could have impacted which sites permitted recruitment ads and who chose to participate (Jahnke, Imhoff, et al., 2015; Ó Ciardha et al., 2022). Likewise, self-reported data on paraphilic sexual arousal may be susceptible to social desirability and impression management (Pedneault et al., 2021) and participants may have under-reported symptoms and arousal due to stigmatization or concerns regarding detection (Lasher & Stinson, 2017; Ray et al., 2010). Self-reported paraphilic sexual arousal may be conservatively interpreted as a minimum or “floor level” of sexual attraction (i.e., MSICs under-reporting intensity of pedohebephilic arousal versus over-endorsing attractions).

Another element of self-report methods of data collection is that, due to underreporting of sexual offending (Cotter, 2021), it is possible that some non-justice system involved MSICs may have engaged in CSA that had not yet come to the attention of law enforcement (Stephens & McPhail, 2019). Thus, the present study refrains from explicitly labelling this sample as “non-offending,” though such might reasonably be inferred. Accurate measurement of *undetected* CSA is challenging due to ethical responsibilities to ensure participant safety and honest responding while adhering to mandatory reporting protocols. Nevertheless, distinguishing community MSICs by detection status offers opportunity to examine differences between those whose behaviour to-date has not resulted in formal justice involvement, versus those have been “caught” for CSA and have since returned to community.

Finally, data collection occurred in the early weeks of the COVID-19 pandemic. Emerging research on the psychological impact of COVID-19 in North America finds that the pandemic correlated with elevated levels of distress, including depression, anxiety, general lifestyle disturbance, destabilization, and isolation (Best et al., 2021; Panchal et al., 2021). Participants may have reported data during a time of increased mental health disturbance wherein existing vulnerabilities may have been negatively exacerbated by the pandemic.

### Directions for Future Research

The current findings offer multiple directions for future research. These results, including the typological profiles of community MSICs, should be replicated to promote credibility, generalizability, and reproducibility. Studies should test the robustness of the typology using alternative design methodologies (e.g., in-person data collection, indirect measures of sexual interest) or applying new analytical approaches. Accessing a larger comparison sample of MSIC-CSA participants may elucidate similarities and differences between men who have already crossed sexual boundaries, versus MSICs who have not acted (and may never act) on their sexual interests toward children. Such an examination would serve to further differentiate between sexual interests and sexual offence behaviours.

Future research also should extrapolate from the typological model to inform evidence-based preventative interventions that meet the dual needs of subjective wellness and objective risk mitigation. The emergence of distinct Low, Moderate, and High Vulnerability groups in community MSICs suggests that clients may present with different need and protective profiles. Therapeutic targets may be prioritized differently in effective secondary intervention approaches. Examining interactions between factors within and across domains, including whether additive or combined factors affect vulnerability and risk profiles, also presents an area for future study.

Recognition of typological groups may facilitate investigations into help-seeking behaviours among community MSICs. The Vulnerability Typology may provide a framework to understand which groups are most likely to seek or utilize services, what issues most compel help-seeking (e.g., mental health or lifestyle concerns, versus sexual interest in children), and what barriers to services are most relevant for each group. In turn, future research can explore ways to enhance service accessibility and availability for community MSICs.

## Data Availability

The data that support the findings in this study are available from the corresponding author, HS, upon reasonable request.

## Related Versions

The first author conducted this research as part of a doctoral dissertation while a student at the University of New Brunswick, Fredericton, NB, Canada (Stewart, 2022).

## Social Media Accounts

*Mary Ann Campbell:* X
